# Gut Microbiota Mediator Level and Its Relation to Disease Activity in Ankylosing Spondylitis

**DOI:** 10.5152/ArchRheumatol.2025.11048

**Published:** 2025-09-01

**Authors:** Hasan Nasrallah, Özlem Altındağ, Mazlum Serdar Akaltun, Mehmet Akif Bozdayı, Mustafa Örkmez, Elif Balbal, Ali Gür

**Affiliations:** 1 Department of Physical Therapy and Rehabilitation, Medicana Avcilar Hospital, Gaziantep, Türkiye; 2 Department of Physical Therapy and Rehabilitation, aziantep University Faculty of Medicine, Gaziantep, Türkiye; 3 Department of Biochemistry, Kilis State Hospital, Kilis, Türkiye

**Keywords:** Ankylosing spondylitis, enzyme-linked immunosorbent assay, gut microbiota

## Abstract

**Background/Aims:**

It is known that the intestinal microbiota plays an essential role in developing many diseases. In this study, the relationship between gut microbiota markers and clinical parameters of ankylosing spondylitis and the effect of drugs on gut microbiota markers were evaluated. The aim of this study is to evaluate the composition of the gut microbiota in individuals with ankylosing spondylitis by comparing it with that of healthy individuals to assess the potential effects of microbial alterations on disease pathogenesis and inflammatory response and to identify differences based on treatment methods.

**Materials and Methods:**

This study included 76 AS patients diagnosed for at least 2 years, aged between 18 and 65 (38 anti-TNF recipients and 38 nonsteroidal anti-inflammatory drug [NSAID] recipients), and 38 age- and sex-matched healthy volunteers. Detailed clinical evaluations were conducted on patients and volunteers. All patients underwent a systematic clinical evaluation in accordance with the diagnostic and follow-up criteria for ankylosing spondylitis. In this context, the modified Schober test was performed to assess the lumbar flexion range of motion, chest expansion was measured with a tape measure, and cervical and thoracolumbar spinal range of motion was evaluated using a goniometer. Additionally, a detailed peripheral joint examination, including all major and minor joints, was conducted to identify peripheral joint involvement. Relevant areas were also assessed for the presence of enthesitis in terms of tenderness and pain. Sacroiliac joint tenderness was examined through direct palpation and provocation tests. Erythrocyte sedimentation rate (ESR) and C-reactive protein (CRP) were measured in patients and volunteers, and Bath Ankylosing Spondylitis Disease Activity Index (BASDAI) and Bath Ankylosing Spondylitis Functional Index (BASFI) scores were also calculated and recorded in the patient group. CD14, CTLA4, CXC16, lipopolysaccharide (LPS), and TLR4 levels were measured in serum samples using the enzyme-linked immunosorbent assay method.

**Results:**

Bath Ankylosing Spondylitis Disease Activity Index and BASFI scores were significantly higher in the NSAI (Non-Steroidal Anti Inflammatory) recipient group than in the anti-TNF recipient group (*P* < .05). C-reactive protein and ESR levels were significantly lower in patients who received anti-TNF therapy than those who received NSAI therapy (*P* < .05). CTLA4, CXC16, LPS, and TLR4 levels were found to be significantly higher in patients receiving NSAI treatment compared to those receiving anti-TNF treatment and the healthy controls (*P* < .05). There were no significant differences between patients and controls concerning CD14 levels (*P* > .05).

**Conclusion:**

This research observed that CRP and ESR levels and disease activity scores in AS patients who received anti-TNF treatment were lower than those in the NSAID treatment group and even closer to the control group. It was believed that the connection between microbiota markers and clinical and inflammatory markers can indicate the pathogenesis of AS, guide treatment follow-up, and help develop new treatment strategies.

**Main Points**

- Microbiota markers, disease activity and inflammation markers were found to be lower in AS patients receiving anti-TNF therapy and healthy controls than in AS patients receiving NSAI therapy.
- When sCD14, CTLA4, CXCL16, LPS and TLR4 values were compared statistically in the patient and control groups, CTLA4, CXCL16, LPS and TLR4 levels were found to be significantly higher in the patient group (P < 0,05).
- It is believed that intestinal microbiota markers may play an important role in inflammation in AS patients and could serve as monitoring parameters in the clinical course of the disease and treatment success.
- In AS patients, microbiota markers have been shown to be associated with disease activity and pharmacological treatment options.

## Introduction

Ankylosing spondylitis (AS) is a progressive inflammatory disease characterized by chronic inflammation primarily affecting the axial skeleton.^1^ In various studies conducted in the white population of Europe and America, the prevalence of AS has been found to range from 0.05% to 0.23%. In a study conducted in Türkiye, the prevalence of AS was found to be 0.14% in a screening of 1436 males.^2^ Ankylosing spondylitis is an autoimmune disease that occurs through complex interactions between genetic predisposition and environmental factors. Despite significant advances in understanding ankylosing spondylitis, its etiology has not yet been fully elucidated. Studies conducted so far have revealed the association of various factors, including genetic predisposition, immune system response, infection, and endocrine abnormalities, with the development of AS.^3^

In the pathogenesis of rheumatoid arthritis, a chronic inflammatory rheumatic disease, the involvement of mediators associated with the gut microbiota such as CD14, lipopolysaccharide, TLR4, CTLA4, and CXCL16 has been demonstrated.^4^ Soluble CD14 (sCD14) is a model recognition receptor structurally expressed on various types of immune cells. After exposure to bacterial endotoxins, monocytes release soluble CD14 (sCD14) through the protease-dependent shedding of the membrane-bound form of CD14 and the direct secretion of the soluble form. Accordingly, increased sCD14 has been associated with gram-negative bacterial sepsis and other conditions related to microbial translocation. Lipopolysaccharide (LPS) is a glycolipid specific to the cell walls of Gram-negative bacteria. LPS is transferred from a CD14/LBP complex to a TLR4/MD-2 membrane receptor complex on the cell surface. This transmembrane signaling activates nuclear factor (NF)-kB, leading to the induction of gene transcription and the release of pro-inflammatory cytokines, such as tumor necrosis factor-alpha, thereby initiating inflammation. TLR4 and CD14 are components of the innate immune system. When TLR4 binds to lipopolysaccharide-binding protein and CD14, it serves as a receptor for bacterial LPS. The binding of LPS to TLR4 activates a comprehensive cellular signaling pathway that induces inflammatory responses, cytokine expression, and secretion. Cytotoxic T lymphocyte antigen (CTLA-4) is a critical regulatory molecule expressed on T cells that plays an important role in inhibiting T cell activation and peripheral tolerance. CTLA-4 negatively regulates T cell functions. CXC chemokine ligand 16 (CXCL16) is a soluble chemokine that functions as an adhesion molecule. CXCL16 regulates inflammation, tissue damage, and fibrosis. In the pathogenesis of another chronic inflammatory rheumatic disease, AS, it has been shown that the gut microbiota plays a significant role, although how it precisely affects the pathogenesis remains not fully understood.^1,5^ Microbial infection acts as a triggering factor for the host’s natural immune system and the development of AS. In patients with AS, significant differences have been found in the gut microbiota, with bacteria such as Porphyromonadaceae and Bacteroidaceae showing notable distinctions compared to healthy controls.^3^ When this study was being planned, it proceeded with the notion that levels of CD14, LPS, TLR4, CTLA4, and CXCL16 associated with the gut microbiota could offer insights into the pathogenesis and treatment monitoring of AS and the aim was to evaluate the levels of these markers in AS patients undergoing various treatments and compare them with those of healthy controls.

## Methods

This study included individuals who had been diagnosed for at least 2 years and had applied to the Physical Medicine and Rehabilitation Clinic of Gaziantep Faculty of Medicine.

Seventy-six AS patients (38 receiving anti-TNF therapy and 38 receiving nonsteroidal anti-inflammatory drug [NSAID] therapy) aged between 18 and 65 years, along with 38 healthy volunteers with matching age and gender distribution, were recruited. An “Informed Consent Form” was obtained from all participants after they were provided with information about the study. This study was conducted in accordance with the 2008 Helsinki Declaration. Ethical approval was granted by the Ethics Committee of Gaziantep University Faculty of Medicine on June 30, 2021, with decision number 2021/158. Written informed consent was obtained from patients. The diagnosis of AS was established based on the Modified New York criteria.^6^ Individuals with any inflammatory disease other than AS, systemic, metabolic, allergic, cardio-pulmonary, malignant, or psychiatric diseases, as well as those who may have communication barriers, those receiving treatment for hyperlipidemia, those who had used antibiotics in the last 6 months, and pregnant women were excluded from the study. The control group consisted of individuals without a diagnosis or family history of inflammatory, systemic, or metabolic diseases, who did not smoke or consume alcohol.

The ESR was measured in millimeters per hour (mm/h) using the fully automated Starrsed Interliner (Starrsed RL, RR Mechatronics, Netherlands) device, which is based on the modified Westergren method.

The serum CRP level was measured in milligrams per liter (mg/L) using the immuno-turbidimetric method on the Beckman Coulter AU5800 autoanalyzer (Japan) with Beckman Coulter commercial kits (Japan).

The levels of sCD14 (FineTest, Cat. No: EH3747, China), CXCL16 (FineTest, Cat. No: EH0105, China), CTLA4 (FineTest, Cat. No: EH0667, China), and TLR4 (FineTest, Cat. No: EH1033, China) in serum samples were measured using the enzyme-linked immunosorbent assay (ELISA) method with commercial kits. These kits perform measurements based on the sandwich ELISA principle. The level of LPS in serum samples was measured using the ELISA method with a commercial kit (FineTest, Cat. No: EU3126, China). This kit performs measurements based on the competitive ELISA principle.

The normal distribution suitability of numerical variables was assessed using the Shapiro-Wilk test. Variables showing normal distribution were expressed as mean ± SD, while those not showing normal distribution were expressed as Median (25th-75th percentile). The comparison of variables showing normal distribution among 3 groups was conducted using ANOVA and LSD tests, and for comparisons between 2 groups, the Student’s *t*-test was employed. For variables not showing normal distribution, the Kruskal–Wallis and Dunn tests were used for comparisons among 3 groups, and the Mann–Whitney *U* test was used for comparisons between 2 groups. The relationships between categorical variables were tested using the chi-square test, while the relationships between numerical variables not showing normal distribution were tested using the Spearman rank correlation coefficient. SPSS 22.0 for Windows (IBM SPSS Corp.; Armonk, NY, USA) was used for the analyses, and *P* < .05 was considered statistically significant.

## Results

In this study, 38 AS patients receiving NSAID treatment, 38 AS patients receiving anti-TNF treatment, and 38 healthy individuals were included. When the demographic data of patients and healthy individuals were examined, there was no statistically significant difference in terms of age, gender distribution, and exercise habits (*P* > .05 for all) (Table 1).

When AS patients receiving NSAID treatment and those receiving anti-TNF treatment were compared in terms of BASDAI and BASFI scores, BASDAI and BASFI scores were found to be statistically significantly higher in patients receiving NSAID treatment (*P* = .029; .034, respectively). In the patient group, ESR and CRP levels were statistically significantly higher (*P* < .001, for both) (Table 2).

When the values of sCD14, CTLA4, CXCL16, LPS, and TLR4 were statistically compared between the patient and control groups, significant differences were found in the CTLA4, CXCL16, LPS, and TLR4 parameters (*P* < .001, for all). However, there was no significant difference in the sCD14 parameter (*P* = .731) (Table 3).

In patients receiving NSAID treatment, no significant correlation was found between ESR, CRP, BASDAI, and BASFI scores and microbiota markers in the correlation analysis (*P* > .05 for all). In the control group, no significant correlation was found between ESR, CRP, BASDAI, and BASFI scores and ARF (Acute Phase Reactants) parameters in the correlation analysis (*P* > .05 for all).

## Discussion

In this study, the levels of CD14, CTLA4, CXCL16, LPS, and TLR4 in AS patients receiving anti-TNF and NSAID treatments were investigated and were compared with those of healthy controls to evaluate their association with inflammation markers and disease activity. Results indicate that microbiota markers are associated with disease activity and pharmacological treatment options in AS patients. In this regard, this study sheds light on elucidating the pathophysiology of AS and monitoring drug treatments.

The disease activity parameters were found to be lower in the anti-TNF treatment group compared to the NSAID and control groups. This finding was interpreted as anti-TNF therapy potentially being more effective than NSAID drugs in reducing disease activity. The results were consistent with the literature.^7-9^

The study results indicated that inflammation markers were similar between the anti-TNF group and healthy controls but lower compared to those receiving NSAID treatment. This outcome is consistent with findings from similar studies.^10^

It is thought that immunological processes in AS may occur due to a lack of protection against microbial pathogens in the gut or changes in intestinal permeability. Studies suggest a relationship between intestinal and joint inflammation, proposing that clinical remission can be achieved with normal intestinal histology.^11^

The intestinal microbiota is a dynamic concept influenced by environmental and nutritional behaviors. It has been shown to play a role in various processes such as the production of bioactive compounds, protection against pathogens, energy homeostasis, nutrient metabolism, and regulation of immunity.^12^ The presence of an inflammatory response and microscopic intestinal inflammation in the intestinal mucosa of 50%-60% of patients with AS suggests an etiopathogenetic link with the microbiota.^13,14^

In a study conducted by Haidmayer et al,^15^ they investigated the role of sCD14 in psoriatic arthritis, a type of spondyloarthritis. They reported that plasma concentrations of sCD14 were not associated with disease activity, intestinal permeability, or intestinal inflammation. Additionally, they found that sCD14 levels did not change after probiotic intake, suggesting that sCD14 levels are independent of the severity of inflammation.^15^ These results indicated that in the anti-TNF group, sCD14 levels were not associated with disease activity scores and inflammation markers. Based on the assumption that AS is a polygenic disease, van der Paardt et al^16^ concluded that CD14 alleles and genotypes were not associated with clinical manifestations of AS. In a similar manner, Luchetti et al^17^ found sCD14 to be insignificant in axial SpA/IBH, peripheral SpA/IBH, IBH, and AS groups compared to the control group.

It has been reported that serum antibody levels against LPS are significantly elevated in patients with ankylosing spondylitis compared to healthy controls, and LPS antibody levels have been reported to be strongly correlated with levels of CRP.^18^ Luchetti et al^17^ suggested that levels of LPS were significantly higher in patients with spondyloarthritis compared to healthy controls. In this study, the levels of LPS were found to be higher in AS patients receiving NSAID treatment compared to AS patients receiving anti-TNF treatment and the control group, which was consistent with the literature findings.

For TLR4, there is a balance between the pro-inflammatory cell surface signaling pathway and the anti-inflammatory intracellular signaling pathway.^19^ It has been reported that TLR4 protein and mRNA levels are higher in AS patients compared to healthy individuals.^20^ Yang et al^20^ reported that TLR4 protein levels were closely linked to serum levels of inflammatory cytokines such as TNF-α and IL-12 and disease activity in AS patients. Assassi et al,^21^ in a clinical study conducted in the American population, found that TLR4 was overexpressed in AS patients compared to controls, and gene expression of TLR4 was significantly reduced after treatment with anti-TNF drugs. In a similar study, it has been reported that TLR4 mRNA is significantly increased in AS patients.^22^ Consistent with these findings, Assassi et al^21^ also found that TLR4 levels decreased with anti-TNF treatment.

In this study, TLR4 levels were found to be significantly higher in AS patients receiving NSAID treatment compared to AS patients receiving anti-TNF treatment and the control group. This result suggests that the suppression of inflammation after anti-TNF treatment leads to a decrease in microbiota marker levels, indirectly indicating the course of the disease.

Toussirot et al^23^ reported that serum CTLA-4 levels in patients with spondyloarthritis are associated with clinical and inflammation parameters. Çetintepe et al^24^ suggested that CTLA-4 levels are high in patients with AS, proposing a potential role for the CTLA-4 molecule in the pathogenesis of AS. The study results revealed that CTLA-4 levels were significantly lower in patients receiving anti-TNF therapy and in the control group compared to those receiving NSAID treatment. There is no controlled clinical study in the literature demonstrating the effect of drug treatments on CTLA-4 levels. In this regard, this study is the first to elucidate the effect of drug treatments on CTLA-4.

These results indicated that CXCL16 levels were higher in AS patients receiving NSAID treatment compared to those receiving anti-TNF therapy and healthy controls.

Yılmaz et al^25^ reported elevated levels of CXCL16 in AS patients. CXCL16 is also a pro-angiogenic chemokine, enhancing angiogenesis, which could shed light on the etiopathogenesis of the disease. These results represent the first study to compare post-treatment levels of CXCL16 with healthy controls.

The study results indicated that microbiota markers, disease activity, and inflammation markers were lower in AS patients receiving anti-TNF therapy and healthy controls compared to those receiving NSAI therapy. However, no positive or negative correlation was found between disease activity, inflammation markers, and microbiota markers. This result could be attributed to the composition of the study group, which consisted of patients who had been diagnosed and under treatment for a long time, and the inability to evaluate the disease in terms of flare-ups and remission periods.

It is believed that intestinal microbiota markers may play an important role in inflammation in AS patients and could serve as monitoring parameters in the clinical course of the disease and treatment success. More controlled clinical research is needed on this subject.

The patient group consisted of individuals who had been diagnosed with AS and had been receiving drug therapy for at least 2 years. One of the limitations is that patients who were newly diagnosed and not receiving any treatment were not included in the study. Assessing the same parameters at different stages of the disease could have provided more informative results. The fact that the results are based on one-time measurements can also be considered a limitation. Additionally, not conducting interviews with patients and controls regarding their dietary habits is another limitation of the study.

Microbial response disorders between women and men may be due to factors such as the body’s interaction with the immune system, hormones, and microbes, in particular. For example, the individual’s relationship system is generally stronger, and this may have different effects on the microbiome. The potential of estrogen to modulate elasticity responses may indicate that women’s responses to microbes differ. Furthermore, metabolic processes of microbes, such as short-chain fatty acids, differ by gender, which may amplify their effects in AS. The microbial response differences between men and women were not evaluated in the study. This is one of the limitations of this study.

This study was designed based on the notion that intestinal microbiota markers may be associated with inflammation in AS and could potentially be linked to treatment outcomes. With this aim, the levels of microbiota markers in AS patients receiving 2 different drug therapies were evaluated, comparing them with clinical and inflammation parameters, and healthy controls.

The results not only revealed the relationship between microbiota markers and inflammation and clinical parameters but also demonstrated that anti-TNF therapy is more effective in controlling inflammation and clinical outcomes compared to other treatments. Anti-TNF therapy suppresses inflammation by inhibiting tumor necrosis factor (TNF)-alpha. Elevated levels of TNF-alpha can particularly lead to an increase in TLR4 and other inflammatory markers. Since anti-TNF therapy blocks this pathway, TLR4, LPS, CXCL16, and CTLA4 levels may be found to be lower as a result. This reflects the anti-inflammatory effect of anti-TNF therapy. Individuals in the control group, who are healthy individuals not receiving treatment, generally have lower levels of inflammatory markers. The fact that their levels are similar to those of the anti-TNF group reflects a situation where there is no treatment effect. This suggests that even healthy individuals not receiving treatment can exhibit similar baseline responses.

As the first clinical study investigating the association between CD14, CTLA4, CXCL16, LPS, and TLR4 levels with disease progression and treatment outcomes in AS patients, it is believed that this study could contribute to the literature.

Considering these results, it may be worthwhile to integrate microbiota marker assessments into clinical practice, as they could offer valuable insights for monitoring treatment responses and potentially inspire the development of novel therapeutic strategies for AS.

## Figures and Tables

**Table 1.** The Demographic Data of Patients and Controls

	The Patient Group Receiving NSAID Treatment (n = 38)	The Patient Group Receiving Anti-TNF Therapy (n = 38)	The Control Group (n = 38)	*P*
Female n (%)	14 (36.8)	15 (39.4)	14 (36.8)	.899
Male n (%)	24 (64.2)	23 (61.6)	24 (64.2)	
Age (year)	41.28 ± 9.43	40.38 ± 8.33	40.05 ± 8.01	.861
Exercise habit (yes/no)	5/33	6/32	5/33	.745

Anti-TNF, anti-tumor necrosis factor; Mean, average; NSAID, nonsteroidal anti-inflammatory drug.

*P* < .05.

**Table 2.** Clinical and Biochemical Parameters in Patients and Controls

Variables	The Patient Group Receiving NSAID Treatment (n = 38)	The Patient Group Receiving Anti-TNF Therapy (n = 38)	The Control Group (n = 38)	*P*
BASDAI	7.29 ± 1.29	4.93 ± 1.93	–	.029*
BASFI	6.08 ± 2.0	4.21 ± 2.4	–	.034*
ESR (mm/h)	28.43 ± 12.31	17.28 ± 11.42	16.23 ± 11.42	<.001*
CRP (mg/L)	12.38 ± 5.17	5.73 ± 4.88	5.83 ± 4.25	<.001*

Anti-TNF, anti-tumor necrosis factor; BASDAI, Bath Ankylosing Spondylitis Disease Activity Index; BASFI, Bath Ankylosing Spondylitis Functional Index; Mean, average; NSAID, nonsteroidal anti-inflammatory drug.

*P < .05.

**Table 3.** The Mean Levels of sCD14, CTLA4, CXCL16, Lipopolysaccharide, and TLR4 in the Patient and Control Groups

Variables	The Patient Group Receiving NSAID Treatment (n = 38)	The Patient Group Receiving Anti-TNF Therapy (n = 38)	The Control Group (n = 38)	*P*
sCD14 (ng/mL)	1.79 ± 0.62	1.71 ± 0.76	1.84 ± 1.17	.731
CTLA4 (ng/mL)	1.18 ± 0.88	0.51 ± 0.88	0.57 ± 0.79	.001*
CXCL16 (ng/mL)	2.85 ± 1.58	1.55 ± 0.57	1.57 ± 0.41	.001*
LPS (µg/mL)	106.44 ± 35.68	69.64 ± 37.95	81.82 ± 42.26	.001*
TLR4 (ng/mL)	2.75 ± 1.44	1.16 ± 0.83	1.49 ± 1.77	.001*

Anti-TNF, anti-tumor necrosis factor; CTLA4, cytotoxic T lymphocyte antigen 4; CXCL16, CXC chemokin ligand 16; LPS, lipopolysaccharide; NSAID, nonsteroidal anti-inflammatory drug; sCD14, soluble CD14; TLR4, toll-like receptor 4.

*P < .05.

## Data Availability

The data that support the findings of this study are available on request from the corresponding author.
